# Development of Sham Yoga Poses to Assess the Benefits of Yoga in Future Randomized Controlled Trial Studies

**DOI:** 10.3390/life11020130

**Published:** 2021-02-07

**Authors:** Ramya Ramamoorthi, Daniel Gahreman, Timothy Skinner, Simon Moss

**Affiliations:** 1 College of Health and Human Sciences, Charles Darwin University, Ellengowan Drive, Darwin 0909, Australia; daniel.gahreman@cdu.edu.au (D.G.); simon.moss@cdu.edu.au (S.M.); 2 Department of Psychology, Center for Health and Society, Copenhagen University, 1050 Copenhagen, Denmark; ts@psy.ku.dk; 3 La Trobe Rural Health School, La Trobe University, Bendigo 3550, Australia

**Keywords:** asanas, cardiovascular activity, diabetes mellitus, metabolic syndrome, yoga

## Abstract

Background: Although research has demonstrated the benefits of yoga to people who have been diagnosed with diabetes or at risk of diabetes, studies have not confirmed these effects can be ascribed to the specific features of the traditional postures, called asanas. Instead, the effects of asanas could be ascribed to the increase in cardiovascular activity and expenditure of energy or to the expectation of health benefits. Therefore, to establish whether asanas are beneficial, researchers need to design a control condition in which participants complete activities, called sham poses, that are equivalent to traditional asanas in physical activity and expectation of benefits. Objectives: The aim of this research was to design an appropriate suite of sham poses and to demonstrate these poses and traditional asanas are equivalent in energy expenditure, cardiovascular response, and expectations of health benefits. Methods: Twenty healthy men at medium to high risk of developing diabetes volunteered to partake in the current study. These men completed two sessions that comprised traditional asanas and two sessions that comprised sham poses—poses that utilize the same muscle groups as the asanas and were assigned fictitious Sanskrit labels. Before and after each session, heart rate, blood pressure, blood glucose levels, triglycerides levels, and oxygen saturation were measured to gauge the intensity of exercise. After each session, using a standard measure, participants also indicated the degree to which they expected the poses to improve health. Results: The degree to which the sessions affected the physiological measures (for example, pre-exercise, the heart rate for yoga and sham was 71.06 ± 4.79 and 73.88 ± 6.05, respectively, and post-exercise, the heart rate was 70.19 ± 6.16 and 73 ± 7.55, respectively) and the expectations of health improvements did not differ between the traditional asanas and the sham poses. Likewise, the degree to which each session influenced these physiological measures was negligible in both conditions. Conclusions: This study developed a series of poses that elicit similar physiological and psychological effect as traditional yoga asanas. These poses can be used in an active control group in future randomized trial studies that are designed to assess the benefits of asanas.

## 1. Introduction

### 1.1. Prevalence of Diabetes

Currently, 415 million people suffer from diabetes worldwide [1]. Furthermore, the prevalence of type 2 diabetes mellitus is projected to rise to 642 million by 2040 [2]. In Australia, for example, type 2 diabetes is the fastest growing chronic condition: an estimated 2.5 to 3 million Australians could potentially develop diabetes by 2031 [3,4]. Given the morbidity, mortality, and economic impact of diabetes, the prevention of type 2 diabetes has become a major global health priority [5].

Fortunately, a diversity of initiatives could be introduced to prevent or to manage the deleterious consequences of diabetes. Indeed, three landmark studies in China, Finland, and the USA [6] demonstrated that diabetes could be substantially delayed, and conceivably prevented, if individuals are supported in introducing and sustaining relatively modest changes to their lifestyle, such as decreasing their dietary fat consumption, increasing their fiber consumption, and increasing their levels of physical activity.

### 1.2. The Benefits of Yoga

One activity that could facilitate the prevention of diabetes is yoga. Yoga is an ancient Indian science and a style of life that incorporates asanas (specific postures), pranayama (breathing practices), dhyana (meditation), mantras (chants), and sutras (wisdom teachings) [7,8,9,10]. Meta-analyses have confirmed the health benefits of yoga interventions in people diagnosed with type 2 diabetes mellitus, although further studies are necessary to overcome the limitations of previous studies and to clarify the underlying mechanisms [11,12]. For example, research has not clarified whether these benefits can be attributed to the immunological, cardiovascular, musculoskeletal, emotional, or other effects of yoga [13,14]

Despite this uncertainty, some compelling studies suggest that yoga is an effective adjunct to standard treatments of type 2 diabetes. For example, Kumar and colleagues demonstrated that yoga, when combined with standard medical treatments, was more likely to reduce fasting blood glucose than standard medical treatments alone [15]. Furthermore, Thind and colleagues showed that yoga did not only diminish blood glucose but also significantly improved lipid profiles and other health parameters [16]. Other studies have shown that three months of yoga reduce oxidative stress and the glycemic index in patients diagnosed with type 2 diabetes [17,18].

### 1.3. The Mechanisms That Underpin These Benefits

Although yoga comprises a coordinated blend of practices and beliefs, many researchers attribute the benefits of this technique merely to the increase in cardiovascular activity and the expenditure of energy [19]. As an exercise, yoga is a low-intensity physical activity that increases metabolism at rates that are slightly higher than resting levels [20]. Research has explored the biochemical changes that such physical activity elicits—especially changes that might ameliorate metabolic syndrome [21]. Some of the positive changes of yoga on fasting blood glucose and other diabetes risk factors such as levels of triglycerides, oxygen saturation, heart rate, and blood pressure in response to yoga can partly be ascribed to increased expenditure of energy. In fact, research has confirmed that expenditure of energy enhances glucose tolerance and insulin action [22].

However, some of these physiological changes in response to yoga interventions could partly be attributed to distinct features of yoga asanas. Practitioners assume the asanas comprise postures that specifically facilitate the endocrine and nervous systems [20]. Indeed, previous researchers have suggested that yoga could be utilized to manage diabetes-induced disorders more effectively than other physical activities [17,18,23,24,25].

To explore this possibility, some research has investigated whether asanas are more beneficial than other physical activities to people afflicted with diabetes or metabolic syndrome. For example, Kanaya and colleagues explored whether 48 weeks of stretching activities were more beneficial to patients diagnosed with metabolic syndrome if complemented with yoga [18]. The stretching exercises significantly diminished fasting blood glucose, insulin, and hemoglobin A1c (HbA1c). The inclusion of yoga, however, did not significantly affect these results. Nevertheless, other characteristics, such as the expected benefits of these exercises, were not controlled, and thus future research is warranted.

### 1.4. Rationale

Yoga therapy research in clinical populations was conducted to show the effect of 3 months of yoga on oxidative stress, glycemic status, and anthropometry in type 2 diabetes mellitus, and Yoga and sham yoga had identical effects on oxidative stress, glycemic status, and anthropometry in type 2 diabetes mellitus [26]. Research has yet to resolve many vital questions around the benefits of asanas to people who experience metabolic syndrome. First, research has not established whether asanas are more effective than other physical activities that are equivalent in physical intensity. Until this research is conducted, the benefits of asanas could merely be ascribed to increases in cardiovascular activity and the expenditure of energy. Consequently, to improve their health, people who experience metabolic syndrome or type 2 diabetes could benefit from comparable physical activity rather than need to learn and practice asanas in particular.

Second, research has not determined whether the benefits of asanas over comparable physical activities could be ascribed to the reputation or credibility of yoga and the expectation of improvement. Arguably, because asanas are deemed as integral to yoga, individuals might assume these postures may be more effective than physical activities that are comparable in intensity. These expectations, and not the postures themselves, might improve health indices, such as glycemic control, lipid profile, heart rate, and blood pressure.

Third, research into the health benefits of yoga has primarily been restricted to individuals who have been diagnosed with type 2 diabetes. This research, therefore, has mainly explored whether yoga could be an adjunct to existing treatment regimes. In contrast, research should also explore whether yoga could help prevent type 2 diabetes. That is, researchers should investigate whether yoga in general, and asanas in particular, might benefit individuals who are deemed to be at risk of diabetes.

To ascertain whether the effects of asanas cannot be merely ascribed to physical activity in general or expectations of health benefits, researchers need to design a suitable control condition. Specifically, they need to design physical activities that are comparable to asanas in both physical intensity and expectations of health benefits. The aim of this study was to develop and validate this control condition, called sham yoga. Sham yoga entails poses that seem to resemble asanas, deploy the same muscle groups as asanas, but diverge from the cardinal asana postures. This study also determined whether sham yoga poses and actual yoga poses are comparable in physical intensity, as measured by cardiovascular responses and energy expenditure, and expectations of health benefits.

## 2. Methods

### 2.1. Study Design and Overview

This study had a repeated-measures design in which participants completed two yoga and two sham-yoga sessions. The order in which participants completed each session was randomized. All participants demonstrated a medium or high risk of developing type 2 diabetes.

Before and after each session, researchers assessed the heart rate, blood pressure, blood glucose levels, triglycerides levels, and oxygen saturation of each participant to gauge the intensity of physical activity. Researchers also measured the degree to which each participant expected the session to improve their health. This study received approval from the Human Research Ethics Committee of the Charles Darwin University (CDU-HREC: HR15106).

### 2.2. Participants

To recruit participants, the first author posted flyers on various locations in the university campus, local community, and shopping center. Furthermore, staff at the university received an invitation, promoting the study. These flyers and emails sought males, above 18 years of age, who performed less than 30 min of moderate or vigorous exercise a week, were not prescribed any medication, and had not participated in yoga sessions previously. Females were excluded to preclude the effects of menses on physiological and psychological measures.

To identify participants at risk of diabetes, individuals who expressed interest in this study completed the Australian type 2 diabetes risk assessment tool, (AUSDRISK). Only participants who exhibited medium or high risk of developing type 2 diabetes mellitus, as defined by AUSRISK, were included. Participants also completed the International Physical Activity Questionnaires to exclude individuals who performed moderate or vigorous exercise more than 30 min a week [27,28,29] (Table S1). Finally, to exclude participants who could not perform the yoga or sham yoga properly, these individuals completed the Physical Activity Readiness Questionnaire [30].

**Figure 1:**
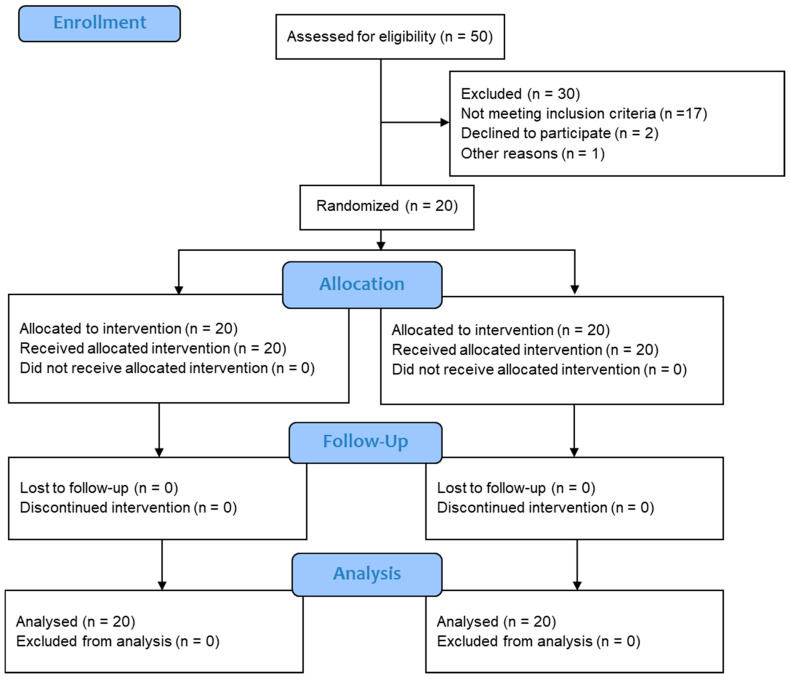
The Consolidated Standards of Reporting Trials (CONSORT) flow diagram indicating the screening and selection of the included studies.

As Figure 1 reveals, 50 men expressed interest in this study; 30 of these men were excluded from the analysis because they were either not eligible, decided to withdraw, or did not complete all four sessions. The final sample comprised 20 men at risk of diabetes, who were otherwise healthy and agreed to participate in this study. A priori power analysis, conducted using STATA/IC 15.0 (StataCorp LLC, College Station, TX, USA), indicated the probability this sample size would detect an effect size of 0.6 or higher exceeding 0.8, assuming a correlation of 0.6 between the two conditions.

### 2.3. Interventions

Every participant completed two yoga sessions and two sham-yoga sessions—each comprising 10 poses. The order in which each participant completed these sessions was randomized. Participants were not informed that two of the sessions comprised sham poses. To control the effect of diet and diurnal variation, training sessions started at 7:00 a.m., and participants had fasted overnight.

The yoga condition comprised 10 asanas—poses that past research indicate may benefit individuals diagnosed with diabetes [31].

Each asana lasted 5 to 6 min. These asanas comprised ardha matsyendrasana (half lord of fishes pose), vajrasana (thunderbolt pose), trikonasana (triangle pose), padhahastasana (hand under foot pose), danurasana (bow pose), kakasana (crow pose), bhujangasana (cobra pose), salabasana (locust pose), pashimotasana (forward-seated bend pose), and savasana (corpse pose). Practitioners assume these asanas target and stimulate the digestive system as well as induce deep relaxation, purportedly addressing both the somatic and the mental origins of diabetes.

**Figure 2:**
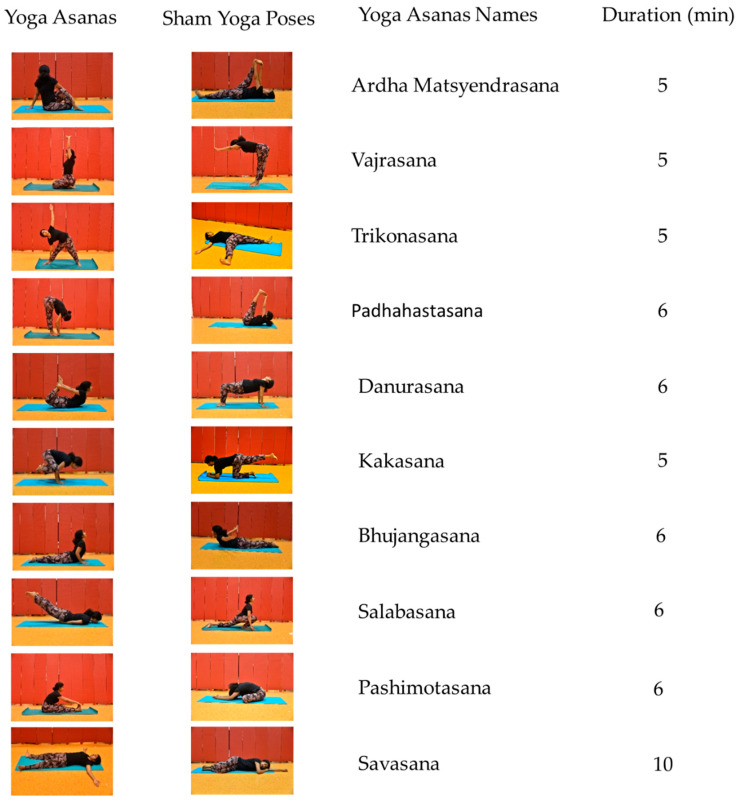
Yoga asanas and the equivalent sham yoga poses with suggested durations.

The sham-yoga condition also comprised 10 poses—poses that superficially resembled the asanas but diverged from traditional practices. These sham poses were also designated fictitious Sanskrit names and introduced to participants by the same yoga instructor. An Exercise and Sport Scientist designed poses that stimulate the same agonist and antagonist muscle groups as the corresponding asanas. Consequently, the intensity of physical activity and the credibility of these behaviors should not differ between the sham poses and traditional asanas. Figure 2 presents a visual image of each asana and the corresponding sham poses.

### 2.4. Outcome Measurements

#### 2.4.1. Anthropometric Parameters

In the first session, the researchers gauged anthropometric measures, including height, weight, waist circumference, and body composition. Bioelectrical impedance analysis technology was utilized to gauge body composition (Tanita Corporation of America, Inc., Arlington Heights, IL, USA).

#### 2.4.2. Assessment of Physiological Parameters

Both before and 3 min after each session, researchers collected a drop of blood from the index finger of each participant. A handheld blood analyzer (Accutrend^®^ Plus, Roche Diagnostics, Indianapolis, IN, USA) was then utilized to ascertain the blood glucose and triglyceride levels. In addition, researchers utilized heart rate monitors (ActiGraph GT9X Link, Pensacola, FL, USA) to determine the heart rate of participants before and immediately after each session. Finally, before and immediately after each session, researchers utilized an automatic monitor (Omron HEM-7130) and a fingertip Oximeter (CONTEC CMS50DL) to gauge blood pressure and oxygen saturation, respectively.

#### 2.4.3. Assessment of Psychometric Properties of Credibility and Expectancy of Yoga and Sham-Yoga Sessions

At the end of each session, participants completed the Treatment Credibility and Expectancy Questionnaire [32]. This measure assesses confidence in the treatment, the willingness of participants to recommend the treatment to a friend, and their belief in the likelihood this treatment will help their condition (Table S2).

### 2.5. Statistical Methods

SPSS 25.0 (SPSS Inc., Chicago, IL, USA) was utilized to conduct a two-way repeated measures Analysis of Variance (ANOVA) to ascertain whether the disparity between pre-session and post-session measures differed between the yoga and the sham-yoga conditions. The probability level of statistical significance was set at *p* ≤ 0.05. The Cohen’s *d* formula was utilized to measure effect sizes [33], with values of 0–0.2, 0.2–0.6, 0.6–1.2, 1.2–2, 2–4 deemed as trivial, small, moderate, large, and very large effects [27].

Because individuals completed two sessions in each condition, Intra-class Correlation Coefficients (ICC) and 95% confident intervals were calculated based on a single-rating (k = 1), absolute-agreement, 2-way mixed-effects model [29]. Based on the 95% confident intervals of the ICC estimate, values less than 0.5, between 0.5 and 0.75, between 0.75 and 0.9, and greater than 0.90 were assumed to signify poor, moderate, good, and excellent reliability, respectively [28].

## 3. Results

### 3.1. Anthropometric Measures of the Participants

To characterize the sample, Table 1 presents the mean value of sociodemographic and anthropometric measures including age, height, weight, body fat, waist circumferences (WC), and AUSDRISK score (Table 1).

### 3.2. Physiological Parameters

Table 2 presents the mean and standard deviation of each physiological measure, such as fasting blood glucose, triglycerides levels, oxygen saturation, heart rate, systolic blood pressure, and diastolic blood pressure, before and after the session—for the yoga and sham conditions, separately. This table also displays the effect sizes, representing the disparity between pre-session and post-session values. Finally, to characterize the degree to which the two yoga sessions—and the two sham sessions—generated comparable values, this table presents the intra-class correlations, 95% confidence intervals of these correlations, and coefficients of variation.

Blood glucose, triglycerides levels, heart rate, blood pressure, and oxygen saturation did not significantly differ between the yoga and the sham conditions. Furthermore, the extent to which these parameters changed from before the session to after the session also did not differ between yoga and sham conditions; that is, none of the interactions were significant. Similarly, expectations of improvement did not differ significantly between the yoga and the sham-yoga sessions.

## 4. Discussion

### 4.1. Key Findings

Despite the purported benefits of yoga to individuals who have been diagnosed with type 2 diabetes, past research has not shown that traditional asanas or postures are indeed necessary. Instead, the purported benefits of asanas could be ascribed to the effects of physical activity—and thus cardiovascular responses and energy expenditure—or expectations of improved health. Therefore, to establish the benefits of asanas, researchers need to develop an active control group that performs poses that are equivalent to asanas in intensity of physical activity and expectations of improvement. Consistent with this aim, past researchers, such as Park and colleagues, have underscored the scarcity of suitable control groups in yoga research [34].

This study was designed to develop and to validate a set of poses that researchers can utilize in a sham control group, primarily to assess the health benefits of traditional asanas. This sham-yoga condition comprised 10 postures. Each posture stimulates the same muscle groups as a corresponding asana and was assigned a fictitious Sanskrit label. Because of these similarities, the sham poses and traditional asanas should be equivalent in intensity and expected health improvements.

To validate these sham poses, 20 men completed two sessions of traditional asanas and two sessions of sham poses in random order. Heart rate, blood pressure, blood glucose levels, triglyceride concentration, and oxygen saturation did not differ between the yoga and the sham conditions. Likewise, the change in these indices during the sessions were modest, or even negligible, and did not differ between these conditions. As these findings indicate, traditional asanas and sham poses expend comparable levels of energy—and thus do not differ in physical intensity. Furthermore, the degree to which individuals expected these movements to benefit their health did not differ between the yoga and the sham conditions. Non-significant effects can, in principle, be ascribed to limited power. Nevertheless, the intra-class correlations attest to the reliability of most physiological measures—a key determinant of power. The intraclass correlation of heart rate and oxygen saturation, however, was lower, potentially diminishing power.

As this study demonstrates, researchers who plan to evaluate the benefits of asanas could utilize these poses in a control group. Furthermore, because the participants in this study demonstrated a medium to high risk of diabetes, these poses may be useful in studies designed to gauge whether these asanas benefit individuals at risk of diabetes. Specifically, if researchers can verify the benefits of asanas in people at risk of diabetes, they will reveal that yoga might be germane to the prevention, rather than merely to the management or treatment, of type 2 diabetes and metabolic diseases.

None of the yoga or sham sessions significantly affected glucose or triglyceride concentrations. However, as past research shows, whether yoga affects blood glucose levels depends on several parameters [35,36]. For example, when the frequency, duration, and intensity of practice increases, yoga is more likely to diminish fasting blood glucose [37,38]. This result implies that asanas may not confer immediate benefits to people at risk of diabetes but might be effective if maintained at a reasonable intensity over an extended period.

### 4.2. Key Limitations and Implications

Before researchers apply the sham yoga poses in Figure 2, several limitations of this study may need to be considered. First, in this study, the participants had received no training in yoga before. These participants were selected because untrained individuals tend to experience more pronounced cardiovascular benefits in response to regular yoga sessions than do trained individuals [39]. Consequently, if the participants are untrained, the study may be more likely to uncover disparities in the expenditure of energy and cardiovascular activity between traditional asanas and sham yoga. Nevertheless, because participants were untrained, they may not have been flexible enough to execute the poses accurately—potentially obscuring the differences between traditional asanas and sham yoga. Future research, therefore, could perhaps replicate this study, but recruit participants who are agile enough to perform the poses as effectively as possible.

Second, the results could be ascribed to limited statistical power. To override this limitation, future studies could arrange more sessions, more participants, and more direct measures of energy expenditure than cardiovascular responses, such as calorimeters.

In short, this study developed and validated a set of sham poses that researchers, when striving to explore the benefits of asanas, could deploy in the control group. Alternatively, when conducting these studies, researchers could measure, and then statistically control, expenditure of energy and expectations of health benefits. Yet, to control these effects statistically, researchers need to know the relationship between the various outcomes and either energy expenditure or expectations of health benefits. Because these relationships are often unknown, this sham control is more likely to be preferable.

## 5. Conclusions

In conclusion, this study achieved designing an equivalent control that matched psychological, physiological, and metabolic factors to yoga. The poses developed in this study could be utilized for future randomized trial studies that investigate the efficacy of yoga over modern training programs.

## Figures and Tables

**Table 1:** Characterization of the participants in the study.

Variables	Mean ± Standard Deviation
Age (years)	44.95 ± 9.80
Height (Centimeter)	175.50 ± 6.89 cm
Weight (kilogram)	88.72 ± 23.13 kg
Body Mass Index (BMI)	27.95 ± 7.17
Bodyfat (percentage)	26.64 ± 7.59%
Waist circumferences (WC) (Centimeter)	104.00 ± 18.98 cm
AUSDRISK Score	12.80 ± 4.00

**Table 2:** Intra-class correlation coefficients of results pre- and post-exercise in yoga and sham-yoga conditions.

		Pre-Exercise					Post-Exercise					Effect Size	
		Mean ± SD	ICC	95% CI		CV%	Mean ± SD	ICC	95% CI		CV%	ES	
Glucose (mmol/L)	Yoga	4.81 ± 1.39	0.805	0.568	0.918	17.9	4.88 ± 1.58	0.827	0.617	0.927	17.4	0.05	Trivial
	Shm	5.11 ± 1.98	0.905	0.875	0.980	13.5	4.93 ± 1.81	0.937	0.849	0.975	10.6	0.10	Trivial
Triglycerides (mmol/L)	Yoga	1.70 ± 0.52	0.582	0.202	0.810	24.2	1.69 ± 0.78	0.446	0.040	0.0733	28.5	0.01	Trivial
	Shm	1.96 ± 0.58	0.291	−0.182	0.647	30.6	1.71 ± 0.52	0.125	−0.336	0.533	39.6	0.47	Small
Oxygen Saturation (%)	Yoga	97.30 ± 0.97	0.012	−0.448	0.452	1.4	97.45 ± 0.67	−0.013	−0.368	0.389	1.0	0.18	Trivial
	Shm	97.18 ± 1.02	181	−0.273	0.570	1.2	97.48 ± 0.88	0.356	−0.102	0.685	0.9	0.32	Small
Heart Rate (bpm)	Yoga	71.06 ± 4.79	0.199	−0.279	0.588	8.1	70.19 ± 6.16	0.262	−0.204	00.627	9.6	0.16	Trivial
	Shm	73.88 ± 6.05	0.420	−0.019	0.722	7.5	73.00 ± 7.55	0.620	0.246	0.831	6.9	0.13	Trivial
Systolic blood pressure (mmHg)	Yoga	122.20 ± 14.82	0.743	0.459	0.889	6.2	123.95 ± 15.00	0.793	0.548	0.912	6.1	0.12	Trivial
	Shm	121.63 ± 13.49	0.643	0.285	0.843	7.4	123.23 ± 13.91	0.435	0.030	0.725	10.1	0.12	Trivial
Diastolic blood pressure (mmHg)	Yoga	83.73 ± 10.12	0.629	0.261	0.836	8.8	82.65 ± 8.96	0.542	0.145	0.789	9.4	0.11	Trivial
	Shm	83.15 ± 10.27	0.411	−0.039	0.719	11.6	84.07 ± 8.94	0.371	−0.077	0.693	10.7	0.10	Trivial

Typical Error as Coefficient of Variation (CV) (%), Effect Size (ES). Shm denotes Sham yoga.

## Supplementary Materials

The following are available online at https://www.mdpi.com/2075-1729/11/2/130/s1, Table S1: IPAQ Questionnaires, Table S2: Psychometric properties questionnaire.

## Abbreviations

CI	Confidence Interval
PAR-Q	Physical Activity Readiness Questionnaire
IPAQ	International Physical Activity Questionnaires
AUSDRISK	Australian type 2 diabetes risk assessment tool
WC	Waist circumference
ANOVA	Analysis of Variance
ES	Effect sizes
RCT	Randomized Control Trials
FBG	Fasting Blood Glucose

## References

[1] Papatheodorou K., Papanas N., Banach M., Papazoglou D., Edmonds M. (2016). Complications of diabetes 2016. J. Diabetes Res..

[2] Ogurtsova K., da Rocha Fernandes J.D., Huang Y., Linnenkamp U., Guariguata L., Cho N.H., Cavan D., Shaw J.E., Makaroff L.E. (2017). IDF Diabetes Atlas: Global estimates for the prevalence of diabetes for 2015 and 2040. Diabetes Res. Clin. Pract..

[3] Diabetes Australia. The Diabetes Australia Research Program. https://www.diabetesaustralia.com.au/wp-content/uploads/Diabetes-the-silent-pandemic-and-its-impact-on-Australia.pdf.

[4] Magliano D.J., Peeters A., Vos T., Sicree R., Shaw J., Sindall C., Haby M., Begg S.J., Zimmet P.Z. (2009). Projecting the burden of diabetes in Australia–what is the size of the matter?. Aust. N. Z. J. Public Health.

[5] Zheng Y., Ley S.H., Hu F.B. (2018). Global aetiology and epidemiology of type 2 diabetes mellitus and its complications. Nat. Rev. Endocrinol..

[6] The Diabetes Prevention Program Research Group (2002). The Diabetes Prevention Program (DPP): Description of lifestyle intervention. Diabetes Care.

[7] Manyam B.V. (2004). Diabetes Mellitus, Ayurveda, and Yoga.

[8] Bijlani R.L., Vempati R.P., Yadav R.K., Ray R.B., Gupta V., Sharma R., Mehta N., Mahapatra S.C. (2005). A brief but comprehensive lifestyle education program based on yoga reduces risk factors for cardiovascular disease and diabetes mellitus. J. Altern. Complementary Med..

[9] Jain S.C., Uppal A., Bhatnagar S., Talukdar B. (1993). A study of response pattern of non-insulin dependent diabetics to yoga therapy. Diabetes Res. Clin. Pract..

[10] Skoro-Kondza L., Tai S.S., Gadelrab R., Drincevic D., Greenhalgh T. (2009). Community based yoga classes for type 2 diabetes: An exploratory randomised controlled trial. BMC Health Serv. Res..

[11] Cui J., Yan J.H., Yan L.M., Pan L., Le J.J., Guo Y.Z. (2017). Effects of yoga in adults with type 2 diabetes mellitus: A meta-analysis. J. Diabetes Investig..

[12] Innes K.E., Selfe T.K. (2016). Yoga for Adults with Type 2 Diabetes: A Systematic Review of Controlled Trials. J. Diabetes Res..

[13] Chong C.S., Tsunaka M., Tsang H.W., Chan E.P., Cheung W.M. (2011). Effects of yoga on stress management in healthy adults: A systematic review. Altern. Ther. Health Med..

[14] Uebelacker L.A., Epstein-Lubow G., Gaudiano B.A., Tremont G., Battle C.L., Miller I.W. (2010). Hatha yoga for depression: Critical review of the evidence for efficacy, plausible mechanisms of action, and directions for future research. J. Psychiatr. Pract..

[15] Kumar V., Jagannathan A., Philip M., Thulasi A., Angadi P., Raghuram N. (2016). Role of yoga for patients with type II diabetes mellitus: A systematic review and meta-analysis. Complementary Ther. Med..

[16] Thind H., Lantini R., Balletto B.L., Donahue M.L., Salmoirago-Blotcher E., Bock B.C., Scott-Sheldon L.A. (2017). The effects of yoga among adults with type 2 diabetes: A systematic review and meta-analysis. Prev. Med..

[17] Hagins M., Rundle A., Consedine N.S., Khalsa S.B.S. (2014). A randomized controlled trial comparing the effects of yoga with an active control on ambulatory blood pressure in individuals with prehypertension and stage 1 hypertension. J. Clin. Hypertens..

[18] Kanaya A.M., Araneta M.R.G., Pawlowsky S.B., Barrett-Connor E., Grady D., Vittinghoff E., Schembri M., Chang A., Carrion-Petersen M.L., Coggins T. (2014). Restorative yoga and metabolic risk factors: The Practicing Restorative Yoga vs. Stretching for the Metabolic Syndrome (PRYSMS) randomized trial. J. Diabetes Its Complicat..

[19] Wolever R.Q., Bobinet K.J., McCabe K., Mackenzie E.R., Fekete E., Kusnick C.A., Baime M. (2012). Effective and viable mind-body stress reduction in the workplace: A randomized controlled trial. J. Occup. Health Psychol..

[20] Govindaraj R., Karmani S., Varambally S., Gangadhar B. (2016). Yoga and physical exercise—A review and comparison. Int. Rev. Psychiatry.

[21] Khatri D., Mathur K., Gahlot S., Jain S., Agrawal R. (2007). Effects of yoga and meditation on clinical and biochemical parameters of metabolic syndrome. Diabetes Res. Clin. Pract..

[22] (2006). Washington University School of Medicine CALERIE Group Improvements in glucose tolerance and insulin action induced by increasing energy expenditure or decreasing energy intake: A randomized controlled trial. Am. J. Clin. Nutr..

[23] McDermott K.A., Rao M.R., Nagarathna R., Murphy E.J., Burke A., Nagendra R.H., Hecht F.M. (2014). A yoga intervention for type 2 diabetes risk reduction: A pilot randomized controlled trial. BMC Complementary Altern. Med..

[24] Gothe N.P., Keswani R.K., McAuley E. (2016). Yoga practice improves executive function by attenuating stress levels. Biol. Psychol..

[25] Gordon L.A., Morrison E.Y., McGrowder D.A., Young R., Fraser Y.T.P., Zamora E.M., Alexander-Lindo R.L., Irving R.R. (2008). Effect of exercise therapy on lipid profile and oxidative stress indicators in patients with type 2 diabetes. BMC Complementary Altern. Med..

[26] Hegde S.V., Adhikari P., Kotian S.M., Shastry R. (2020). Effects of Yoga Versus Sham Yoga on Oxidative Stress, Glycemic Status, and Anthropometry in Type 2 Diabetes Mellitus: A Single-Blinded Randomized Pilot Study. Int. J. Yoga Ther..

[27] Hopkins W.G. A New View of Statistics. http://sportsci.org/resource/stats/.

[28] Koo T.K., Li M.Y. (2016). A Guideline of Selecting and Reporting Intraclass Correlation Coefficients for Reliability Research. J. Chiropr. Med..

[29] McGraw K.O., Wong S.P. (1996). Forming inferences about some intraclass correlation coefficients. Psychol. Methods.

[30] Canadian Society for Exercise Physiology Physical Activity Readiness Questionnaire—PAR-Q. https://www2.fgcu.edu/mariebcollege/RS/files/EIM_PAR_Q1.pdf.

[31] Ramamoorthi R., Gahreman D., Skinner T., Moss S. (2019). The effect of yoga practice on glycemic control and other health parameters in the prediabetic state: A systematic review and meta-analysis. PLoS ONE.

[32] Devilly G.J., Borkovec T.D. (2000). Psychometric properties of the credibility/expectancy questionnaire. J. Behav. Ther. Exp. Psychiatry.

[33] Lakens D. (2013). Calculating and reporting effect sizes to facilitate cumulative science: A practical primer for t-tests and ANOVAs. Front. Psychol..

[34] Park C.L., Groessl E., Maiya M., Sarkin A., Eisen S.V., Riley K., Elwy A.R. (2014). Comparison groups in yoga research: A systematic review and critical evaluation of the literature. Complementary Ther. Med..

[35] Sharma M., Knowlden A. (2012). Role of yoga in preventing and controlling type 2 diabetes mellitus. J. Evid. Based Complementary Altern. Med..

[36] Yang K. (2007). A review of yoga programs for four leading risk factors of chronic diseases. Evid. Based Complementary Altern. Med..

[37] Singh S., Malhotra V., Singh K.P., Madhu S.V., Tandon O.P. (2004). Role of yoga in modifying certain cardiovascular functions in type 2 diabetic patients. J. Assoc. Physicians India.

[38] Yogendra J., Yogendra H.J., Ambardekar S., Lele R.D., Shetty S., Dave M., Husein N. (2004). Beneficial effects of yoga lifestyle on reversibility of ischaemic heart disease: Caring heart project of International Board of Yoga. J. Assoc. Physicians India.

[39] Tyagi A., Cohen M. (2016). Yoga and heart rate variability: A comprehensive review of the literature. Int. J. Yoga.

