# Supplementary material for: Development of Sham Yoga Poses to Assess the Benefits of Yoga in Future Randomized Controlled Trial Studies
Source: Life (Basel). 2021 Feb 7;11(2):130. doi: 10.3390/life11020130 (PMC7915153; doi:10.3390/life11020130)
Supplement: Supplementary file 1 [file life-11-00130-s001.pdf]

# Supplementary

We are interested in finding out about the kinds of physical activities that people do as part of their everyday lives. The questions are about the time you spend being physically active in the last 7 days. They include questions about activities you do at work, as part of your house and garden work, to get from place to place, and in your spare time for recreation, exercise, or sport. Your answers are important. Please answer each question even if you do not consider yourself to be an active person.

**Table S1.** IPAQ Questionnaires.

1a. During the last 7 days, on how many days did you do vigorous physical activities like heavy lifting, digging, aerobics, or fast bicycling? Think about only those physical activities that you did for at least 10 min at a time.
__________ Days per week _ None (Go to question 2a).
1b. How much time in total did you usually spend on one of those days doing vigorous physical activities?
In answering the following questions:
__________ hours __________ minutes
2a. Again, think only about those physical activities that you did for at least 10 min at a time. During the last 7 days, on how many days did you do moderate physical activities like carrying light loads, bicycling at a regular pace, or tennis? Do not include walking.
__________ days per week _ None (Go to question 3a).
2b How much time in total did you usually spend on one of those days doing moderate physical activities?
__________ hours __________ minutes
3a During the last 7 days, on how many days did you walk for at least 10 min at a time? This includes walking at work and at home, walking to travel from place to place, and any other walking that you did solely for recreation, sport, exercise, or leisure.
__________ days per week _ None (Go to question 4).
How much time in total did you usually spend walking on one of those days?
__________ hours __________ minutes
4. The last question is about the time you spent sitting on weekdays while at work, at home, while doing course work, and during leisure time. This includes time spent sitting at a desk, visiting friends, reading travelling on a bus, or sitting or lying down to watch television.
During the last 7 days, how much time in total did you usually spend sitting on a week day?
__________ hours __________ minutes

Psychometric properties of the credibility and expectancy questionnaire: We would like you to indicate below how much you believe, right now, that the therapy you are receiving will help you reduce your anxiety. Belief usually has two aspects to it: (1) what one thinks will happen and (2) what one feels will happen. Sometimes these are similar; sometimes they are different. Please answer the questions below. In the first set, answer in terms of what you think. In the second set answer in terms of what you really and truly feel. We do not want your therapist to ever see these ratings, so please keep the sheet covered when you are done.

**Table S2.** Psychometric properties questionnaire.

In the first set, answer in terms of what you think.
Set I
1. At this point, how logical does the therapy offered to you seem?

1 2 3 4 5 6 7 8 9

not at all logical somewhat logical very logical

2. At this point, how successfully do you think this treatment will be in reducing your trauma symptoms?

1 2 3 4 5 6 7 8 9

not at all useful somewhat useful very useful

3. How confident would you be in recommending this treatment to a friend who experiences similar problems?

1 2 3 4 5 6 7 8 9

not at all confident somewhat confident very confident

4. By the end of the therapy period, how much improvement in your trauma symptoms do you think will occur?

0% 10% 20% 30% 40% 50% 60% 70% 80% 90% 100%

In the second set, answer in terms of what you really and truly feel.

Set II

For this set, close your eyes for a few moments and try to identify what you really feel about the therapy and its likely success. Then answer the following questions.

1. At this point, how much do you really feel that therapy will help you to reduce your trauma symptoms?

1 2 3 4 5 6 7 8 9

not at all somewhat Very much

2. By the end of the therapy period, how much improvement in your trauma symptoms do you really feel will occur?

0% 10% 20% 30% 40% 50% 60% 70% 80% 90% 100%
